# Examining rural-urban disparities in perceived need for health care services among adults with disabilities

**DOI:** 10.3389/fresc.2022.875978

**Published:** 2022-08-09

**Authors:** Gilbert Gimm, Catherine Ipsen

**Affiliations:** ^1^Department of Health Administration and Policy, George Mason University, Fairfax, VA, United States; ^2^Rural Institute for Inclusive Communities, University of Montana, Missoula, MT, United States

**Keywords:** rural, health care, unmet need, perceived need, preventive services, disability

## Abstract

**Purpose:**

The purpose of this study is to parse out differences between unmet need and perceived need for health care services among rural and urban adults with disabilities in the United States. While unmet need focuses primarily on environmental factors such as access to health insurance or provider availability, perceived need relates to personal choice. This distinction between unmet and perceived need is largely ignored in prior studies, but relevant to public health strategies to improve access and uptake of preventive care.

**Methods:**

Using Wave 2 data from the National Survey on Health and Disability, we explored rural and urban differences in unmet and perceived health care needs among working-age adults with disabilities for acute and preventive services.

**Findings:**

Although we found no significant differences in unmet needs between rural and urban respondents, we found that perceived needs for dental care and mental health counseling varied significantly across geography. Using logistic regression analysis and controlling for observable participant characteristics, we found that respondents living in noncore counties relative to metropolitan counties were more likely to report not needing dental care (OR 1.89, *p* = 0.028), and not needing mental health counseling services (OR 2.15, *p* ≤ 0.001).

**Conclusion:**

These findings suggest additional study is warranted to understand perceived need for preventive services and the levers for addressing rural disparities.

## Introduction

Timely access to preventive care such as regular check-ups, health screenings, immunizations, and dental care can lower the risk of developing health complications, identify emerging health issues, reduce the need for aggressive interventions, and lower overall health care costs (1–3). Despite these substantial advantages, however, many people choose to forego preventive care, particularly in rural places (4–6).

The decision to obtain preventive care depends on a variety of environmental and personal factors. Environmental factors are well cited in the literature, and include the cost of preventive care services, local availability of providers, health insurance coverage, as well as travel time and distance from a person's home to a provider facility to receive care (2, 7, 8). Many environmental barriers to services can be addressed through policy and funding initiatives, such as Affordable Care Act provisions to lower the out-of-pocket cost of preventive services (9). However, less straightforward are personal factors that influence decision making, such as trust of medical providers, ideology, self-reliance, and other behavioral norms (4, 10, 11). If patients do not perceive a need for preventive care, some policy efforts to increase access, such as expanding provider availability, may have limited effectiveness.

The purpose of this study is to parse out differences between unmet need and perceived need in a population of rural and urban adults with disabilities. While unmet need focuses primarily on environmental factors, perceived need relates to personal choice. This distinction is largely ignored in prior studies, but relevant to public health strategies to improve access and uptake of preventive care.

## Background

In comparison to adults living in urban areas of the US, rural adults experience significant health disparities. In part, these disparities can be explained by a greater proportion of older adults (65 years or more) living in rural areas (12). After controlling for age, however, rural adults still experience higher rates of chronic conditions, mortality, and disability across the life span (12–14). Socio-economic factors play a role in disparities, such as lower median income, lower educational attainment, and higher poverty rates that impact access to services (15, 16). The literature documents economic-based considerations for delaying or not seeking services, such as high out-of-pocket costs and a lack of insurance coverage, which has a disproportionate impact on rural adults (8, 16).

Limited availability of rural health service providers also comes into play. In comparison to urban areas, rural areas generally have fewer per capita health care providers including physicians, surgeons, psychologists, counselors, dentists, dieticians, occupational therapists, physical therapists, and a host of allied-health professionals (16, 17). Although telemedicine has been touted as a solution to address provider shortages, rural people have significantly lower rates of broadband access, smartphones, and home computers, as well as an under-trained workforce for using telemedicine visits (16). When health care professionals are unavailable or alternatives such as telemedicine are inappropriate, rural residents incur additional opportunity costs to access care, such as securing reliable transportation and taking time off to travel to health care appointments (16, 18). In international studies, a lack of reliable transportation and financial barriers also contribute to disparities in accessing health care services for adults with disabilities (19).

Rural and urban disparities may also relate to differences in behavioral norms. Prior studies have shown lower rates of seat belt use and higher rates of smoking in rural relative to urban areas (10, 20). Higher rates of smoking undermines the argument that cost by itself is a driving factor in rural decision-making. Rural adults also have lower rates of vaccination, which has become particularly evident during the COVID-19 pandemic. In this case, public health efforts and free COVID-19 vaccinations nationwide have not translated into similar vaccination rates for adults in rural versus urban areas (21, 22).

A study exploring rural and urban differences in worker's compensation healthcare claims provides additional evidence regarding different rural and urban behaviors. After controlling for demographics, injury, and injury severity, Young et al. found that rural workers used significantly fewer physical therapy (PT) services than their urban counterparts, and experienced longer injury duration and risk of prolonged work-disability (11). Although access to PT services may drive some of these differences, cost was not a factor because worker's compensation covered medical costs.

We know that health behaviors shape and directly influence health outcomes (23). In the case of rural adults, less adherence to recommended preventive health practices translates into higher rates of health conditions, comorbidities, and mortality (10, 20). A reluctance to engage in preventive practices is particularly risky for people with disabilities, who often experience a thin margin of health (24). For instance, a recent study of National Longitudinal Survey of Youth 79 (NLSY79) data found that U.S. adults who experience a mobility impairment at age 40 had a higher incidence of health conditions or complications (e.g., arthritis, heart problems, depression, ulcers, intestinal problems, tooth and gum trouble, chronic lung disease, thyroid issues, asthma, diabetes, etc.), compared with those who did not report a mobility impairment. Furthermore, rates of health conditions or complications were significantly higher among rural people for many conditions (Ipsen et al., in press). For this reason, we are particularly interested in exploring rural and urban differences in unmet and perceived need for adults with disabilities. Also, prior studies have not been able to measure or assess perceived need using national surveys, which is a major gap in the literature. Therefore, this study aims to contribute new knowledge on differences in perceived and actual need among adults with disabilities living in rural and non-rural areas of the United States.

## Methods

We used data from the second administration (Wave 2) of the National Survey on Health and Disability to explore unmet and perceived health care needs among people with disabilities. The NSHD was developed as part of a National Institute on Disability, Independent Living, and Rehabilitation Research (NIDILRR) funded grant to learn how access to health insurance and services affects health and community living outcomes among people with disabilities.

### Sample inclusion criteria

Data collection for Wave 2 of the NSHD was completed in February 2020, just before the start of COVID-19 lockdowns in the United States. The sample included adult respondents (*N* = 2,161) who were living in the United States, between the ages of 18 and 64 years, and answered yes to the question “Do you have a physical condition, mental illness, impairment, disability or chronic health condition that can affect your daily activities OR that requires you to use special equipment or devices, such as a wheelchair, walker, TDD, or communication device?” Most participants completed the NSHD questionnaire using an online Qualtrics survey, but all eligible participants had the option of completing the survey over the telephone, if needed.

Recruitment of participants took place in two ways. Targeted recruitment occurred through (1) direct email requests to past respondents who provided optional email contact information, (2) flyers distributed to conference attendees at six 2019 disability-focused conferences, and (3) recruitment materials shared through 73 disability-related organizations, groups, and/or service providers. Additional recruitment was conducted through an online platform called Amazon's Mechanical Turk (MTURK).

MTurk is an online marketplace where “requesters” can post a human intelligence task (HIT), such as completing a survey, and “workers” can pick up the HIT for a small payment. MTurk recruitment began with a brief online screening survey to identify respondents meeting inclusion criteria, who were then invited to take the full survey. To ensure data quality, researchers used vetted strategies including the use of MTurk approval ratings, cognitive checks, and hidden screening criteria to reduce false reporting (25–27). More complete descriptions of NSHD data-collection methods are available in past study publications (26, 27).

### Measures

The NSHD is a comprehensive survey that covers multiple topics including health status, transportation, use of personal assistance services, community participation and social isolation, benefits, employment, insurance coverage, unmet needs, income, demographics, and multiple measures of disability. Survey measures specific to this paper are described below.

#### Socio-demographics

We used several variables to control for socio-demographic differences, including age, gender, race/ethnicity, educational attainment, marital status, and income. *Gender* options included male, female, and other (non-binary). The question for *race and ethnicity* asked, “which one or more of the following best describe your race and/or ethnicity?”: American Indian/Native American, African American/Black, Asian, Hispanic, Native Hawaiian/Pacific Islander, White/Caucasian, and Other. These data were used to create African American/Black, Hispanic, Multi-Racial, and Other Race groups, with the remaining classified as White/not Hispanic. We collapsed seven *education* groups ranging from no formal education to graduate or doctoral degree into four including high school or less, some college, 4-year college, and graduate school for our analyses. The survey asked, “what is your current *marital status*?” with four possible responses: single-never married, single-divorced or widowed, married, and prefer not to answer. *Income* groupings were calculated based on three questions including number of people living in the household, state of residence, and household income. This information was utilized to provide household income as percent of the Federal Poverty Level (FPL). As informed by created variables within the NSHD dataset, we focused on four income categories including < 138% of the FPL, 138–249% of the FPL, 250–399% FPL, and 400% or higher than the FPL.

#### Disability

We measured disability using the 6-item question set included in the American Community Survey (ACS), which includes the following yes/no questions: Are you deaf or do you have serious difficulty hearing? Are you blind or do you have serious difficulty seeing even when wearing glasses? Because of a physical, mental, or emotional condition, do you have serious difficulty concentrating, remembering, or making decisions? Do you have serious difficulty walking or climbing stairs? Do you have difficulty bathing or dressing? Because of a physical, mental, or emotional condition, do you have difficulty doing errands alone, such as visiting a doctor's office or shopping? (28). In our analyses, individuals who endorsed more than one functional difficulty were classified as having multiple disabilities.

#### Noncore, micropolitan, and metropolitan residence

The NSHD asked participants to provide their county of residence. County indicators were matched to Federal Information Processing Standards (FIPS) codes and classified using the Office of Management and Budget's (OMB) rural-urban classification scheme. Specifically, OMB classifies counties as (1) *metropolitan*, if they are located within an urban core of 50,000 of more people, or are an outlying county with close economic ties to an urban core (2) *micropolitan*, if they are located within an urban core of at least 10,000 but < 50,000 people, and (3) *non-core*, all remaining counties (29). Both micropolitan and non-core counties are considered rural, but have unique characteristics related to health care access (30).

#### Unmet need and perceived need

The NSHD asked a series of questions to understand unmet need in the past 12 months for those with some type of health insurance plan. We focused on unmet needs related to a participant's self-reported access to doctors, specialists, prescription medications, dental services, and mental health counseling. NSHD questions on unmet need were phrased as follows: “in the past 12 months, have you been able to [see the doctors you need to; see the specialists you need to; get all the prescription medications you need; get the dental services you need; get the mental health services and/or counseling services you need]. There were four possible response options including “yes,” “no,” “I don't know,” and “I did not need.” While the “no” responses to questions on access to care reflected unmet need, the “I did not need” responses were used to measure and examine differences in perceived need.

### Data analyses

Data was transferred from Qualtrics to STATA for analyses. We compared unadjusted responses to questions about unmet need by geographic area (i.e., non-core, micropolitan, metropolitan) using Chi-square statistics. We then used logistic regression to adjust for observable characteristics and examine factors significantly associated with a participant responding “I did not need” dental services or mental health services. We focused on these two services because they represented proxies for preventive care, as opposed to a measure of acute care from a primary care doctor or specialist, or medications obtained from a pharmacy to manage a chronic health condition.

## Results

Our analytic sample included a total of 2,161 adult respondents (18–64 years) with disabilities. Of these, 6.3% were living in non-core rural areas, 11.1% were in micropolitan rural areas, and 82.6% were living in metropolitan urban areas. Table 1 reports sample demographics including age, gender, race/ethnicity, educational attainment, marital status, income level, and functional disability type by geographic area, and for the combined sample.

**Table 1:** Participant characteristics.

	**Non-core** ***N* = 136)**	**Micropolitan** **(*N* = 240)**	**Metropolitan (*N* = 1,785)**	**Full sample** **(*N* = 2,161)**
Age (mean years)	41.3	42.4	41.6	41.7
**Gender**				
Male	39.0%	31.7%	32.6%	32.7%
Female	58.8%	67.5%	64.0%	64.1%
Other (Non-binary)	2.2%	0.8%	3.4%	3.0%
**Race/Ethnicity**				
White non-hispanic	83.0%	87.3%	79.7%	80.7%
African American	3.0%	2.5%	5.3%	4.8%
Hispanic	2.2%	0.4%	3.4%	3.1%
Multi-Racial	5.9%	6.8%	6.7%	6.6%
Other Race	5.9%	3.0%	4.8%	4.7%
**Educational attainment**				
High school or less	23.0%	18.8%	11.9%	13.3%
Some college	43.0%	49.2%	36.4%	38.3%
4-year college	26.7%	21.3%	27.7%	26.9%
Graduate education	7.4%	10.8%	24.0%	21.5%
**Marital status**				
Currently married	36.1%	35.9%	34.3%	34.6%
Living with partner	11.3%	13.9%	10.4%	10.8%
Divorced/widowed	14.3%	15.6%	14.1%	14.3%
Never married	38.4%	34.6%	41.3%	40.3%
Income level*				
less than 138% FPL	49.6%	43.2%	35.1%	37.0%
138%-249% FPL	24.1%	26.3%	22.0%	22.6%
250%-399% FPL	14.3%	14.8%	19.5%	18.6%
400% FPL or more	12.0%	15.7%	23.4%	21.9%
Functional disability type**				
Hearing only	1.5%	3.3%	4.0%	3.8%
Vision only	3.7%	3.3%	4.2%	4.1%
Cognitive only	32.4%	27.5%	26.9%	27.3%
Ambulatory only	18.4%	21.8%	17.8%	18.3%
Self-care only	4.4%	2.9%	4.4%	4.2%
Independent living only	26.5%	19.6%	18.7%	19.3%
Multiple disabilities	24.3%	28.0%	27.9%	27.7%

* FPL, federal poverty level.

** Disability type based on binary indicators from 6 categories in the American Community Survey.

### Unmet and perceived needs

Table 2 shows the unadjusted prevalence of unmet and perceived service needs by geographic area (non-core, micropolitan, and metropolitan). There were no statistical differences between non-core, micropolitan, and metropolitan respondents in terms of rates of unmet and perceived need for seeing a doctor, seeing a specialist, and getting needed prescriptions. However, perceived need, as measured by the “I did not need” responses, were statistically different for dental care and mental health care across non-core, micropolitan, and metropolitan groups. For dental care, 17.9% of non-core respondents indicated they did not need dental services, compared to 14.1% in micropolitan, and 9% in metropolitan. For mental health services, 46.7% of non-core, 43.5% of micropolitan, and 34.8% of metropolitan respondents said that they did not need mental health services.

**Table 2:** Unmet and perceived service need (%), by geographic category.

**In the last 12 months have you been able to…?**	**Non-core** **(*N* = 136)**	**Micropolitan** **(*N* = 240)**	**Metropolitan** **(*N* = 1,785)**	**Chi-square** ***p*-value**
**See a doctor**				
Yes	81.3	85.1	83.9	0.571
No	13.1	12.9	12.8	
Did not need to see a doctor	5.6	2.0	3.3	
**See a specialist**				
Yes	76.1	70.4	75.1	0.378
No	11.9	12.2	12.6	
Did not need to see a specialist	11.9	17.3	12.2	
**Get needed prescriptions**				
Yes	77.4	80.5	77.2	0.803
No	16.0	15.1	17.6	
Did not need prescriptions	6.6	4.4	5.2	
**Get dental services**				
Yes	50.0^a^	55.2^a^	60.6^a^	**0.008**
No	32.1^a^	30.7^a^	30.4^a^	
Did not need dental services	17.9^b^	14.1^a, b^	9.0^a^	
**Get counseling or mental health services**				
Yes	34.3^a^	38.9^a^	45.0^a^	**0.024**
No	19.0^a^	17.6^a^	20.3^a^	
Did not need mental health services	46.7^a^	43.5^a, b^	34.8^b^	

Each subscript letter (a, b) denotes a subset of 3 category population density categories whose column proportions do not differ significantly from each other at a 0.05 level.

Boldface indicates p-values < 0.05.

### Logistic regression models to predict perceived need

Given the unadjusted disparities observed in the perceived need for dental care and mental health services among non-core, micropolitan, and metropolitan respondents, we conducted multivariate analyses of these two outcomes. Table 3 reports results from a logistic regression to examine the likelihood of a participant saying they did not need dental care. After controlling for observable participant characteristics, we found that adults in non-core areas (OR 1.89, *p* =0.028) had significantly greater odds of not needing dental care compared to those living in urban areas (i.e., the reference group). Males (OR 2.07, *p* ≤ 0.001) relative to females, individuals from lower income brackets (<138% FPL, OR 2.11, *p* = 0.007 and 138%-249% FPL, OR 1.93, *p* = 0.020), relative to the highest income bracket, and those reporting ambulatory disability (OR 2.22, *p* ≤ 0.001) were also more likely to say they did not need dental care. Conversely, adults were less likely to say they did not need care as they become older (OR 98, *p* =0.044).

**Table 3:** Multivariate analysis of dental care not needed (*N* = 1,705).

	**OR**	***p*-value**	**95% CI**	
**Geographic region (ref** **=** **metropolitan**)				
Non-core	1.89	**0.028**	1.07	3.33
Micropolitan	1.58	0.059	0.98	2.53
Age (Years)	0.98	**0.044**	0.97	1.00
**Gender (ref**. **=** **female)**				
Male	2.07	**<0.001**	1.47	2.92
Other (non-binary)	1.30	0.636	0.44	3.84
**Race/Ethnicity (ref**. **=** **white, non-Hispanic)**				
African American	0.63	0.316	0.26	1.54
Hispanic	0.38	0.190	0.09	1.61
Multi-Racial	0.76	0.452	0.37	1.56
Other Race	1.37	0.418	0.64	2.91
**Education (ref**. **=** **4-year college)**				
High school or less	1.32	0.315	0.77	2.26
Some college	1.07	0.752	0.71	1.62
Graduate school	0.70	0.196	0.40	1.20
**Marital status (ref**. **=** **never married)**				
Currently married	1.12	0.593	0.73	1.72
Living with unmarried partner	0.80	0.485	0.43	1.50
Divorced / widowed / separated	1.23	0.458	0.71	2.12
**Household income (ref**. **=** **400% FPL or more)**				
<138% FPL	2.11	**0.007**	1.22	3.62
138%-249% FPL	1.93	**0.020**	1.11	3.35
250%-399% FPL	1.41	0.250	0.79	2.53
**Functional disability type**				
Hearing only	0.77	0.596	0.29	2.02
Vision only	1.27	0.561	0.56	2.87
Cognitive only	1.01	0.961	0.67	1.52
Ambulatory only	2.22	**<0.001**	1.43	3.44
Self-care only	0.36	0.062	0.13	1.05
Independent living only	1.02	0.940	0.66	1.58
Multiple disabilities only	0.76	0.278	0.46	1.25

Boldface indicates p-values < 0.05.

Table 4 reports results from a logistic regression analysis that examines the likelihood of a participant saying they did not need mental health counseling in the past year. Adults in both non-core (OR 2.15, *p* < 0.01) and micropolitan (OR 1.56, *p* < 0.05) areas had higher odds of saying they did not need mental health services compared to those in metropolitan areas. Males (OR 1.03, *p* ≤ 0.001), relative to females, and respondents as they became older (OR 1.03, *p* < 0.001) were also more likely to report not needing mental health care. Conversely, non-binary adults (OR 0.21, *p* = 0.011), relative to females, were less likely to say they did not need mental health services, as were respondents in the lowest income bracket (138% FPL, OR 0.65, *p* = 0.011), relative to the highest income bracket. In terms of disability, those with a vision disability (OR 2.00, *p* < 0.05) or ambulatory disability (OR 1.37, *p* < 0.05) had higher odds of saying they did not need mental health counseling, while those with a cognitive disability (OR 0.27, *p* < 0.001) and multiple disabilities (OR 0.63, *p* < 0.01) had lower odds of saying they did not need mental health services.

**Table 4:** Multivariate analysis of mental health care not needed (*N* = 1,694).

	**OR**	**p-value**	**95% CI**	
**Geographic region (ref**. **=** **metropolitan)**				
Non-core	2.15	**0.001**	1.37	3.37
Micropolitan	1.56	**0.011**	1.11	2.20
Age (Years)	1.03	**<0.001**	1.02	1.04
**Gender (ref**. **=** **female)**				
Male	1.43	**0.002**	1.14	1.81
Other (non-binary)	0.21	**0.011**	0.06	0.70
**Race/Ethnicity (ref**. **=** **white, non-Hispanic)**				
African American	0.72	0.239	0.41	1.25
Hispanic	1.06	0.858	0.55	2.04
Multi-Racial	0.89	0.610	0.58	1.38
Other Race	1.49	0.159	0.85	2.61
**Education (ref**. **=** **4-year college)**				
High school or less	1.42	0.077	0.96	2.09
Some college	0.90	0.454	0.68	1.19
Graduate school	0.84	0.250	0.62	1.13
**Marital status (ref**. **=** **never married)**				
Currently married	0.87	0.300	0.66	1.14
Living with unmarried partner	1.12	0.561	0.76	1.65
Divorced / widowed / separated	0.90	0.563	0.63	1.28
**Household income (ref**. **=** **400% FPL or more)**				
<138% FPL	0.65	**0.011**	0.47	0.91
138%-249% FPL	0.75	0.086	0.54	1.04
250%-399% FPL	1.26	0.153	0.92	1.73
**Functional disability type**				
Hearing only	1.29	0.348	0.76	2.21
Vision only	2.00	**0.010**	1.18	3.41
Cognitive only	0.27	**<0.001**	0.20	0.37
Ambulatory only	1.37	**0.041**	1.01	1.85
Self-care only	0.71	0.201	0.42	1.20
Independent living only	0.86	0.344	0.62	1.18
Multiple disabilities only	0.63	**0.002**	0.47	0.84

Boldface indicates p-values < 0.05.

## Discussion

The literature highlights the importance of regular health and preventive services for managing health care costs and outcomes (1–3), as well as disparities in health care access and utilization across rural and urban subgroups (6–8). Disparities in access and utilization appear to translate into higher rates of chronic health conditions, mortality, and disability for rural, relative to urban populations (12–14).

The literature points to several rural barriers to health care access including provider shortages, increased out-of-pocket costs, and time and distance to receive services (16–18). We anticipated these types of barriers would be captured by NSHD questions focused on unmet needs in the last 12 months for doctors, specialists, prescription medications, dental services, and mental health counseling. Contrary to expectations, however, unmet needs were similar across non-core, micropolitan, and metropolitan respondents.

When exploring the data further, we noted rural and urban differences in perceived need for services in the last 12-months related to dental and mental health counseling services, after controlling for differences in socio-demographic and disability characteristics. Dental and mental health counseling services are different from doctors, specialists, and prescriptions on certain dimensions. First, they have historically been excluded from most private health insurance plans. According to Fair Health, an advocacy organization for health care costs and coverage, most private health care plans do not cover dental services (31). Although coverage expanded for mental health counseling services somewhat due to the Affordable Care Act, prior to 2014 these types of services were often excluded from coverage (32, 33).

Because rural consumers experience higher rates of poverty, lower household incomes, and less disposable income to cover out-of-pocket costs, lack of current and past insurance coverage for dental and mental health services may contribute to the comparatively low ratios of per capita providers for these services (33). According to the National Center for Health Workforce Analysis, the lowest urban to rural ratios among 32 health care occupations were for dentists (0.61) and psychologists (0.45) (17). In this case, the perception of need may be shaped by long-standing provider shortages that have undermined both demand and expectations for receiving services.

Second, many dental services such as regular teeth cleanings and mental health counseling services to address health behaviors and depression are considered preventive, as opposed to generalized, acute care, which may shape overall consumption (34, 35). Douthit et al., conducted a literature review on rural health care access and highlighted various dimensions of health seeking behaviors. Among these dimensions were cultural perceptions related to accessing care, including delaying care until acute need (36), issues related to privacy when living in a smaller community, and health care consumption, in general (33). As one qualitative study excerpt highlighted “We have our ways. We're from a ranch…We don't use medical. We fix ourselves here” (37).

In particular to mental health counseling, evidence suggests self- and public-stigma undermines decision-making to seek services, particularly in rural communities (38). For instance, a study by Hammer et al. found that self-stigma about seeking counseling was significantly higher among rural men, relative to urban men across diverse socio-demographic backgrounds (39).

Together, these factors may shape decision making differently in rural and urban places, particularly related to perceived need. If preventive health care is not part of existing community norms or typical behavior, perceived need will likely be lower. It is likely that community behaviors develop over time, and environmental factors play a role. If providers are not available and time and cost burdens are high, low demand gets incorporated into the status quo. Goldberg et al. describes this in terms of “horizontal” communication, where people receive and incorporate behaviors based on examples from trusted and familiar sources including personal relationships, social networks, and communities (40).

These findings have important policy implications. Since most federal surveys with questions on health care access are limited to binary “Yes/No” categories without a third option for “not needing a service,” a participant might indicate that they were able to access the care they needed (because they did not perceive or have a need). As a result, the estimated prevalence of having adequate access to care is likely to be misleading or overestimated. The addition of a third response category for “not needing a service” in federal surveys would be a valuable contribution by allowing researchers and policymakers to identify perceived need as a separate component and obtain more accurate measures of access to care.

Further study is needed about which personal and environmental factors play a key role in the lower rates of perceived need for dental care and mental health counseling among adults in rural areas compared to those living in urban areas. Findings from these future studies can also help inform public health efforts to increase the use of preventive care and raise vaccination rates in rural areas.

## Study limitations

This study had several data limitations. First, the data were collected using online survey methods. Thus, the NSHD sample excluded rural and urban adults who had limitations in digital literacy or inadequate broadband access. As a result, these findings may not be generalizable to all adults with disabilities who are living in non-metro, micropolitan, and metropolitan areas. Second, the cross-sectional design of the study using Wave 2 of the NSHD does not allow us to make casual inferences. Instead, we can only identify associations between measures. Third, the NSHD used self-reported measures of access to care in the past 12 months, which may be subject to some recall bias and inaccurate responses.

However, a major strength of the NSHD is that it is a national survey that provides detailed information on health insurance and access to services among working-age adults with disabilities in the United States. Another strength is a novel response option that allows a participant to indicate their perceived need for a service. Other federal surveys only have a binary indicator (yes/no) in response to questions on access to services, which limits the ability to measure perceived need. To our knowledge, this is the first national study to assess perceived need for preventive services among working-age adults with disabilities.

## Conclusion

Preventive services are vital to health management and health outcomes, particularly for adults with disabilities who experience higher rates of chronic health conditions. Unfortunately, preventive services are not consumed at similar rates by geographic area, leaving rural people with disabilities particularly vulnerable to negative health outcomes. We found strong evidence that the perceived need for dental services and mental health counseling was lower among adults in rural areas compared to those living in urban areas. Therefore, perceived need plays a role in examining the demand for preventive health services and highlights the strategic importance of considering differences in community values and norms when developing and implementing public health campaigns. While environmental barriers such as cost and provider availability influence unmet need, rural-urban differences in community norms and expectations also affect the demand for preventive services.

## Data availability statement

The data analyzed in this study are subject to the following licenses/restrictions. These data are available upon request: University of Kansas Institute for Health and Disability Policy Studies, 2022. The National Survey on Health and Disability (NSHD) https://ihdps.ku.edu/nshd. Requests to access these datasets should be directed to Noelle Kurth, pixie@ku.edu; https://ihdps.ku.edu/nshd.

## Ethics statement

The studies involving human participants were reviewed and approved by the University of Kansas Institutional Review Board. Written informed consent for participation was not required for this study in accordance with the national legislation and the institutional requirements.

## Author contributions

GG organized the database and performed the statistical analysis. CI wrote the first draft of the manuscript. CI and GG contributed to conception, design of the study, contributed to all sections of the manuscript, and contributed to manuscript revision, read, and approved the submitted version.

## Funding

This work was supported by the National Survey on Health and Disability (NSHD) as part of the Collaborative on Health Reform and Independent Living (CHRIL). CHRIL was funded by a 5-year Disability and Rehabilitation Research Program (DRRP) from the National Institute on Disability, Independent Living, and Rehabilitation Research (NIDILRR, grant number 90DP0075-01-00). In addition, research for this manuscript was supported by the Research and Training Center on Disability in Rural Communities (RTC:Rural) under another NIDILRR (grant number 90RTCP0002-01-00). NIDILRR is a Center within the Administration for Community Living (ACL), Department of Health and Human Services (HHS). The research does not necessarily represent the policy of NIDILRR, ACL, or HHS, and one should not assume endorsement by the federal government.

## Conflict of interest

The authors declare that the research was conducted in the absence of any commercial or financial relationships that could be construed as a potential conflict of interest.

## Publisher's note

All claims expressed in this article are solely those of the authors and do not necessarily represent those of their affiliated organizations, or those of the publisher, the editors and the reviewers. Any product that may be evaluated in this article, or claim that may be made by its manufacturer, is not guaranteed or endorsed by the publisher.
